# Effects of additional team-based learning on students’ clinical reasoning skills: a pilot study

**DOI:** 10.1186/s13104-017-2614-9

**Published:** 2017-07-14

**Authors:** Meike Jost, Peter Brüstle, Marianne Giesler, Michel Rijntjes, Jochen Brich

**Affiliations:** 1 Department of Neurology and Neuroscience, Medical Center–University of Freiburg, Breisacher Strasse 64, 79106 Freiburg, Germany; 2 Center of Competence for the Evaluation of Teaching in Medicine Baden-Württemberg, Albert-Ludwigs-University Freiburg, Breisacher Strasse 153, 79110 Freiburg, Germany

**Keywords:** Team-based learning, Key feature problem examination, Clinical reasoning, Clinical decision-making, Undergraduate, Neurology

## Abstract

**Background:**

In the field of Neurology good clinical reasoning skills are essential for successful diagnosing and treatment. Team-based learning (TBL), an active learning and small group instructional strategy, is a promising method for fostering these skills. The aim of this pilot study was to examine the effects of a supplementary TBL-class on students’ clinical decision-making skills.

**Methods:**

Fourth- and fifth-year medical students participated in this pilot study (static-group comparison design). The non-treatment group (n = 15) did not receive any additional training beyond regular teaching in the neurology course. The treatment group (n = 11) took part in a supplementary TBL-class optimized for teaching clinical reasoning in addition to the regular teaching in the neurology course. Clinical decision making skills were assessed using a key-feature problem examination. Factual and conceptual knowledge was assessed by a multiple-choice question examination.

**Results:**

The TBL-group performed significantly better than the non-TBL-group (p = 0.026) in the key-feature problem examination. No significant differences between the results of the multiple-choice question examination of both groups were found.

**Conclusions:**

In this pilot study participants of a supplementary TBL-class significantly improved clinical decision-making skills, indicating that TBL may be an appropriate method for teaching clinical decision making in neurology. Further research is needed for replication in larger groups and other clinical fields.

## Background

Since making a neurological diagnosis is often considered complex [1], simply imparting knowledge about neurological diseases or the relevant neuroanatomical background is not enough for teaching neurology: Students need to learn, understand and utilize concepts for diagnosing and treating neurological diseases and apply this knowledge to clinical cases [2, 3]. These clinical skills—often referred to as “clinical reasoning”—are complex mental processes, which need integration und processing of information and drawing conclusions [4, 5]. Of the many different models existing for clinical reasoning, the recently-proposed dual-process theory for clinical reasoning provides a theoretical framework for the integration of two different—but closely-associated and oscillating—systems (Type 1: intuitive pattern recognition, mainly based on clinical experience, and Type 2: analytical thought processes) [5]. The existing methods for teaching these clinical reasoning skills are very heterogeneous and evidence about how to teach clinical reasoning best is still limited [4, 6]. Previous studies have demonstrated positive effects of problem-based learning (PBL) approaches on clinical reasoning skills [7]. Since PBL is a resource intensive approach [8] that cannot be implemented in all medical schools, other teaching strategies like team-based learning (TBL) come into focus. According to Parmelee et al. [9] “TBL is an active learning and small group instructional strategy that provides students with opportunities to apply conceptual knowledge through a sequence of activities with individual work, teamwork and immediate feedback”. TBL is characterized by three key components: (1) individual advance student preparation, (2) individual and team readiness assurance tests and (3) decision-based application assignments in teams [9]. A recent review on the effectiveness of TBL on learning outcomes in health professions education could demonstrate improvement in the domain of factual and conceptual knowledge [10]. However, there is a lack of studies that measure possible effects of TBL on the improvement of clinical reasoning. One method for assessing clinical reasoning is the key feature problem examination [11–13]. A key feature problem consists of a clinical case scenario followed by 3–4 key features that focus only on the critical steps in the solution of a specific clinical problem. Key features also focus on steps in which examinees are most likely to make errors in the solution of the problem and have to capture difficult aspects of practical problem—identification and management. Key feature problem examinations have proved to be a reliable and valid approach in assessing clinical decision-making skills [3, 14–16]. They focus mainly on the Type 2 system of the dual-process theory for clinical reasoning, which is often used by novices (students), but Type 1 system approaches are also possible to solve the questions.

To the best of our knowledge no analysis of the effects of TBL on clinical reasoning skills can be found in the literature. Following the concept of constructive alignment [17], we thought that TBL as an active learning activity might be a good candidate for improving clinical reasoning skills. The aim of this pilot study was therefore to examine if the performance of participants of a supplementary TBL class in a key feature problem examination differs from the performance of students in a non-TBL-class.

We hypothesized that teaching with TBL optimized for instructing clinical reasoning additionally to the regular teaching in seminars would improve key feature problem examination results.

## Methods

### General context

The Neurology Course at the Department of Neurology and Neuroscience at the Medical Center of the University of Freiburg, Germany, usually takes place during the students’ fourth or fifth year of study and is the first contact with clinical neurology. The mandatory 3-week block course includes disease-oriented lectures (12 × 1.5 h, groups of 80 students), symptom-oriented seminars (4 × 1.5 h, groups of 20 students), practical teaching of the neurological examination (2 × 3 h, groups of 6 students) and neurological bedside teaching (7 × 3 h, groups of 6 students). The course ends with a summative multiple choice question examination for all participants covering all course sections.

### Design of the study

**Fig. 1:**
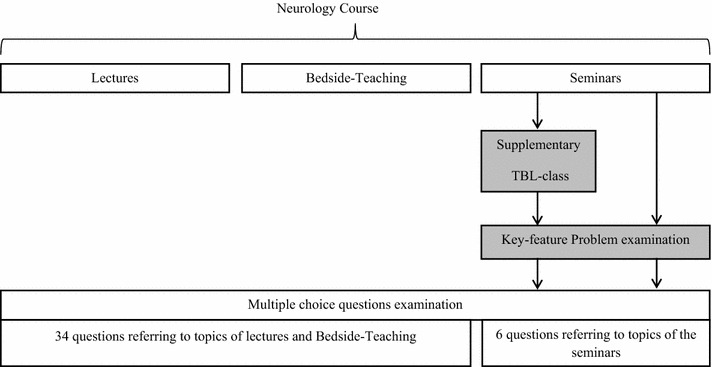
Study design

The Ethics Committee of the Medical Center of the University of Freiburg, Germany, approved our study and all students participating in the key feature problem examination provided written, informed consent. The study was performed using a static-group comparison design: the TBL-class covering the topics of the seminars was offered as a voluntary supplementary class for all participants of the neurology course. The key feature problem examination was offered as a voluntary formative examination to all participants of the TBL-class (TBL-group) and to all students of the neurology course not participating in the TBL-class (non-TBL-group). The summative multiple choice question examination was mandatory for all participants of the neurology course (Fig. 1).

### Sample

Of the 123 students attending the neurology course in the winter semester 2012/2013.

28 students applied for the TBL-class. Due to the tight schedule of students’ fourth or fifth year of study at our university only 17 students (10 fourth-year and 7 fifth-year students; 7 male and 10 female) could participate at the selected time points of the voluntary supplementary TBL-class. The non-TBL-group consisted of 15 students from the same neurology course (10 forth-year and 5 fifth-year students, 6 male and 9 female) with 11 of them having also applied for the TBL-class but could not participate due to their individual schedule (see above).

### Seminars

The seminars with groups of a maximum of 20 students took place in the regular context of the neurology course. The 90-min units covered the topics “vertigo”, “back pain”, “first epileptic seizure” and “acute altered mental status”. The seminars were held interactively and included case-based teaching of specific diseases, forcing students to recollect knowledge about relevant neuroanatomical background and utilize concepts for diagnosing and treating these diseases. Voluntary individual advance preparation was not requested but made possible since the slides used in the seminars were available in advance via the university’s learning management system. The seminars were mandatory for all students, but local study regulation allowed an authorized absence of one seminar. Only long-time experienced board-certified neurologists with postdoctoral lecture qualification acted as teachers for all seminars.

### Team-based learning

The following description of the TBL activities used in this study is based on the proposed guidelines by Haidet et al. [18]. The TBL-units lasted 90 min each. There was one trained instructor (J.B.) for all TBL units who had been teaching TBL for 2 years in voluntary settings [19]. The teams were randomly distributed into groups of five to seven participants. The permanent assignment for all units without possibility of switching was pointed out to all participants. Since TBL is not used in other fields at our Medical School the first TBL-unit about neuroanatomical localization utilizing previous knowledge about neuroanatomy was held to introduce the new teaching method. Subsequently one TBL-unit to each of the above named topics of the seminars was taught.

Preparation (Phase 1) was recommended. Students were asked to read the corresponding seminar slides that were available in advance via the university’s learning management system. Each TBL unit (Phase 2) began with a paper-based, 5-min individual Readiness Assurance Testing, which consisted of three multiple-choice questions on clinical presentations, diagnostics, and therapy. These questions were constructed to cover important issues of clinical reasoning. Each question was subsequently discussed for 5 min in teams. The teams were then responsible for generating an answer and appointing a team spokesperson (team Readiness Assurance Testing). After a prearranged signal, the teams simultaneously held up their answers on colored paper sheets. A discussion moderated by the instructor started among the spokespersons about the different team responses during which the elimination of the alternative answers needed to be actively justified. The instructor gave immediate oral feedback during and after the discussion, which lasted about 10 min and ended with a short summary of the underlying concepts. The application exercises (Phase 3) were each comprised of a clinical case closely based on real cases (“significant problem”) with one to two related multiple-choice questions. The answering options were diagnostic and therapeutic steps, so that the teams had to come up at first with a preliminary diagnosis in order to solve the questions. One application exercise per unit was given to all of the teams to be worked on and discussed for 5–10 min within the teams (“same problem”). Afterwards all teams reported their group results to the audience when given the signal (“specific choice” and “simultaneous reporting”). The teams discussed the selected as well as discarded answers among themselves supervised by the instructor. After the discussion the instructor provided immediate oral feedback and also gave a brief summary of the underlying concepts if necessary pointing out the critical steps in clinical reasoning. No grading or peer evaluations were conducted.

### Key-feature problem examination

The key feature problems were developed with regards to the contents of the seminars according to the steps recommended by Page et al. [11]. They were written by didactically and clinically experienced neurologists of the Department of Neurology and Neuroscience at the University Medical Center Freiburg and reviewed and adapted by two physicians with long-term clinical expertise in neurology who were not involved as authors. Diseases discussed in the TBL application exercises were excluded. The key feature problem examination was intended as a voluntary, formative examination at the end of the neurology course that took place 5 days before the summative multiple-choice examination. Motivation for taking part in the key feature problem examination was encouraged by offering a book voucher as a reward.

The key feature problem examination was conducted in the faculty’s computer lab using a computer-based exam system [20]. Each participant was assigned a unique login and password. Each key feature question could only be answered once, as the correct answer was mostly implied by the following item. Therefore, backward navigation was only possible to review information, not for editing. All answers were centrally recorded on the system’s server. After a short introduction to the test procedure and the test tool, the students had to answer 13 key feature problems in 60 min (four to each of the three seminars “back pain”, “first epileptic seizure” and “acute altered mental status” and five to the seminar “vertigo”). Each problem consisted of three to four key features. As a total, the students had to answer 51 key features, 25 in short menu question format and 26 in long menu format [14, 21]. Each key feature was scored one point, with partial credits of equal weights if not all correct answers were given.

### Multiple choice question examination

The multiple choice question examination (MCQE) closing the neurology course consisted of 40 Type-A multiple choice questions with a set of five options each testing factual or conceptual knowledge. Six questions referred to the four topics addressed in the seminars and TBL units, the remaining 34 questions referred to complementary topics of the lecture and the bedside-teaching, such as multiple sclerosis, dementia, muscle diseases, neuro-oncology and the clinical neurological examination. Three experienced neurologists reviewed all questions internally.

### Statistical analysis

Item analyses were computed for key feature problem examination and MCQE, using Cronbach’s α to determine its internal consistency. Differences between the TBL- and the non-TBL-group were tested by means of t tests for the normally distributed results of the key feature problem examination (verified with the Kolmogorov–Smirnov test) and Mann–Whitney-U tests for non-normally distributed results of the MCQE. Effect size was calculated using Cohen’s d. All statistical analyses were performed with SPSS, version 21 (IBM).

## Results

### Key feature problem examination

Eleven of the 17 TBL-class-participants (8 forth-year and 3 fifth-year students, 5 male and 6 female) completed the key feature problem examination together with the 15 non-TBL-class participants as a non-treatment group. In the post-exam review one key feature problem was excluded from further analysis because of a distinct negative total-item-correlation. The resulting final key feature problem examination consisted of 12 key feature problems (4 to each seminar) with altogether 47 key features—22 of them in the short menu question format and 25 in the long menu format. Cronbach’s α for the key feature problem examination was 0.63. Overall performance of all 26 participating students was 26.2 out of 47 points.

The 11 TBL-class-participants scored a mean of 28.0 points. The 15 students of the control group scored a mean of 24.9 points (Table 1). This difference was statistically significant (p = 0.026) (Table 1). The calculated effect size (Cohen’s d) was 0.84.

**Table 1:** Results of the key feature problem examination and MCQE for the TBL-group and the non-TBL-group

	No of items	TBL-group Mean (SD) Median (IQR) (Min/Max), n = 11 for KFP examination, n = 11 for MCQE	Non-TBL-group Mean (SD) Median (IQR) (Min/Max), n = 15 for KFP examination, n = 13 for MCQE	p value
Key feature problem examination	47	28.0 (4.19)	24.9 (3.59)	0.026
		27.5 (4.88)	25.4 (5.04)	
		(19.6/33.6)	(18.3/31.4)	
Multiple choice question examination	40	35.0 (2.57)	36.0 (2.45)	0.303
		34.0 (3.50)	37.0 (2.00)	
		(31/39)	(30/39)	
Questions referring to topics of seminar/TBL	6	5.4 (0.67)	5.5 (0.66)	0.473
		5.0 (1.00)	6.0 (1.00)	
		(5/6)	(5/6)	
Questions not referring to topics of seminar/TBL	34	29.6 (2.73)	30.5 (2.22)	0.518
		29.0 (4.50)	31.0 (3.00)	
		(25/33)	(25/33)	

*SD* standard deviation, *IQR* interquartile range, *Min* minimum, *Max* maximum

### MCQE

Cronbach’s α for the MCQE was 0.67. Overall performance of the 24 students participating in the key feature problem examination (2 students of the control group did not participate in the MCQE because of being sick at the time point of the MCQE) was 35.5 out of 40 points compared to 34.5 of 40 points of the remaining 97 students of the neurology course (p = 0.193).

There was no statistically significant difference between the TBL-group and the non-TBL-group in the overall mean score as well as in the two subgroups of question referring to the 4 topics of the seminars/TBL (six questions) and to the other neurological topics (34 questions) (Table 1).

## Discussion

This pilot study investigated the effects of a supplementary TBL-class on clinical reasoning skills of fourth- and fifth-year medical students in neurology. Although the number of participants in our pilot study was limited, the students of TBL-group and the non-TBL-group participating in the key feature problem examination matched reasonably well with respect to sex and level of education. Both groups’ performance in clinical decision-making, a result of clinical reasoning, was assessed by a key feature problem examination. The level of factual and procedural knowledge was assessed by a multiple-choice question examination. Both TBL-and non-TBL-groups showed equal overall results in the multiple-choice questions referring to other neurological topics indicating no major differences in neurological knowledge. The multiple-choice questions referring to the content of the seminars were also equally scored by both groups indicating well prepared students in the seminars’ topics in both groups. In contrast, students participating in the TBL-class achieved key feature problem examination scores that were significantly higher than those of the students who fulfilled their neurology course without the additional TBL-class. Key feature problem examinations have been proven to be a reliable and valid approach for the assessment of clinical decision making skills [12, 14, 22] and to represent a feasible tool in evaluating these skills in clinical courses [23, 24]. In this study, using 12 key feature problems with a total of 47 key features in a 60-min examination we were able to achieve an acceptable reliability.

A recent review on the effectiveness of TBL demonstrated an improvement in knowledge scores measured by examinations testing for factual and conceptual knowledge, for example with multiple choice questions [10]. In a previous pilot study on the introduction of the TBL method we could also find an improvement in factual and conceptual knowledge levels measured by a multiple choice question examination [19]. In this previous study the TBL-units were designed to accompany the disease-oriented lectures focusing on repetition and consolidation of knowledge by repeating facts and details from the lecture.

For this study we had students who were well prepared with factual and conceptual knowledge since they mandatorily had to attend the seminars to the four topics which were intensive small group teaching units held by experienced clinical teachers. We therefore optimized the TBL-units for the instruction of clinical reasoning and applied several modifications: The questions of the readiness assurance tests were carefully constructed to focus on important steps in clinical reasoning guiding the way to concepts of structured approaches for solving clinical problems. Emphasis was placed on the presentation of a prototypical clinical case utilized as application exercise to provide an example of a structured clinical reasoning approach suitable for demonstrating analytical strategies of clinical reasoning. The TBL method itself is then an ideal teaching format to stimulate peer discussion about the clarification of crucial steps and pitfalls moreover enabling the instructor to easily identify misunderstandings or misconceptions and correcting them immediately. Taken together this package led to a significantly better result of the TBL-group in the key feature problem examination.

To our knowledge this is the first report demonstrating that TBL can be successfully used to teach clinical reasoning. This is an interesting finding, since evidence-based methods for teaching clinical reasoning are not well established. PBL approaches have demonstrated positive effects on clinical reasoning skills [7, 25], other experts recommend demonstrating multiple clinical cases [3, 6] or the use of human patient simulation [26]. Moreover, teaching clinical reasoning is thought to function best with a clinical experienced, didactically well-trained instructor [6]. In contrast TBL is a highly standardized and cost-effective teaching approach allowing one instructor—who has to be a content-expert but needs no expertise in group processes—to handle up to 200 students simultaneously arranged in teams [9].

Furthermore, other positive effects of TBL are reported for increased motivation of students, better communication processes and team-based skills [27, 28] making TBL an attractive and—for students and instructors—relatively easy to learn and easy to implement teaching approach.

There are several limitations to this study. A major limitation is the comparison of the results of one group with an additional structured intervention to another without this intervention, resulting in an additional 4.5 h of teaching (=18%). Effects in the key feature problem examination may be responsible to the amount of extra time even if the results in the MCQE did not show any differences. Further studies are needed to compare the TBL intervention group directly with an additional instruction time using some other format. We tried to attenuate this effect by using the regular seminar slides as preparation materials available for both groups and used in the seminars by all students. Furthermore, since the results of the TBL-group and the non-TBL-group in the multiple-choice questions referring to the four topics of the seminars/TBL were similar and implicated an equal level of factual or conceptual knowledge, we could demonstrate a significant difference only in the clinical decision making-specific key feature problem examination implicating that the supplementary TBL-units did significantly improve clinical decision making.

Unfortunately we could not recruit more participants for the voluntary participation because of students’ tight schedules. Due to self-selection as method of recruitment students participating in the TBL-class were probably above average motivated. However, since 11 members of the non-TBL-group had initially applied for participation in the TBL class, this proposed higher intrinsic motivation was relevant for the recruitment of the non-TBL-group as well, attenuating a possible bias between the two groups.

Although the experimental and comparison groups performed comparably well in the multiple choice questions referring to other neurological topics indicating the same level of academic performance, some inherent differences might exist between the two groups. For example their clinical experiences might differ because of the completion of different clinical electives, leading to different knowledge and reasoning skills. A pre–post-intervention key feature problem examination design could be used to test for this possible difference, but since neurology is taught at only this particular time in our curriculum we assumed that the participants of our study did not differ much in their previous neurological knowledge and experience. The internal consistency of the key feature problem examination could be improved by additional key-feature problems for each of the training problems, but in order to keep the voluntary test feasible we selected 13 key-feature problems to be able to limit the time of the examination to 60 min. Key feature problem examinations represent a feasible tool for the assessment of students’ clinical decision making skills used in high-stakes examinations and indirect evidence for a correlation with clinical performance does exist insofar that for example the performance in the “Medical Council of Canada Qualifying Examinations Part I” in Canada, which contains a key feature problem examination, is a significant predictor of quality-of-care [29]. But it still remains unclear whether an increase in key feature problem examination scores is related to a direct increase in clinical performance. Studies addressing the effects of TBL optimized for clinical reasoning using performance measures such as the mini-clinical examination exercise (mini-CEX) [30] are required. The study was conducted in one medical school and in one clinical field so the results cannot be generalized. The TBL-class length of five units of 90 min each did not allow the 40 h optimum amount of time to develop highly functional teams that Michaelsen has noted [31], which might have reduced the impact of TBL.

## Conclusion

Taken together we provide first evidence that teaching clinical reasoning with TBL in undergraduate students in a neurology course is effective for improving the clinical reasoning skills as measured by key feature problem examinations. Since this is the first report demonstrating that TBL is a suitable method for teaching clinical reasoning other studies are needed for replication of these results in other and larger students’ populations and other clinical fields.

## Abbreviations

TBL: team-based learning
PBL: problem-based learning
MCQE: multiple-choice question examination
SD: standard deviation
IQR: interquartile range
Min: minimum
Max: maximum
